# Mediterranean Diet Adherence and Ultraprocessed Food Consumption in Relation to Body Composition and Muscle Function Outcomes among Midlife and Older Women: NHANES 2007–2018

**DOI:** 10.1016/j.cdnut.2026.107726

**Published:** 2026-05-26

**Authors:** Bhavana Soma, Angelina Baric, Jennifer Heisz, Alison Shea, Kirsten Bell, Eurídice Martínez Steele, Anthea Christoforou

**Affiliations:** 1 Department of Kinesiology, McMaster University, Hamilton, ON, Canada; 2 Department of Obstetrics and Gynecology, McMaster University, Hamilton, ON, Canada; 3 Department of Nutrition, School of Public Health, University of São Paulo, São Paulo, Brazil; 4 Center for Epidemiological Research in Nutrition and Health instead of Center for Epidemiological Studies in Health and Nutrition, São Paulo, Brazil

**Keywords:** ultraprocessed foods, Mediterranean diet, diet quality, body composition, adiposity, muscle mass, bone mineral density, menopause, NHANES, women’s health

## Abstract

**Background:**

Midlife is a critical period for women’s health, during which hormonal changes increase the risk of unfavorable body composition and functional decline. Diet quality is a key modifiable determinant, yet evidence in midlife and older women remains fragmented, particularly regarding food processing and whole-diet patterns.

**Objectives:**

This study aimed to examine associations of Mediterranean diet adherence and ultraprocessed food (UPF) consumption with body composition and muscle function among midlife and older women, and to assess whether associations differ by menopausal status.

**Methods:**

We analyzed data from a cross-sectional sample of 8376 females aged 45 to 80 y participating in the 2007–2018 NHANES. Dietary intake was assessed using two 24-h recalls, from which Mediterranean diet adherence was estimated using a modified Mediterranean Diet Score, and UPF intake was quantified as the proportion of total grams from NOVA-classified UPFs. Outcomes included BMI, total body fat percentage, android-to-gynoid fat ratio, appendicular lean mass (ALM) relative to BMI (ALM/BMI), grip strength, bone mineral density, and categorical BMI and bone health classifications. Survey-weighted regression models were used with multivariable adjustment. Effect modification by menopausal status was evaluated using interaction terms.

**Results:**

In fully adjusted models, a higher modified Mediterranean Diet Score was associated with lower BMI, total and central adiposity, and greater ALM/BMI. Higher UPF consumption showed opposite associations and was additionally associated with weaker grip strength. Associations with bone outcomes were weak and did not persist after full adjustment. No statistically significant interactions with menopausal status were observed.

**Conclusions:**

Among midlife and older United States women, higher diet quality and lower UPF consumption are associated with more favorable body composition and muscle function, whereas associations with bone health are less evident. These findings highlight the relevance of diet quality and food processing for body composition changes during aging in women.

## Introduction

Women undergo profound physiological changes during midlife, driven largely by the hormonal shifts of the menopausal transition [1,2]. Declines in estrogen and progesterone alter lipid metabolism, insulin sensitivity, and musculoskeletal homeostasis [[3], [4], [5]], contributing to increases in central adiposity, reductions in appendicular lean mass (ALM), and accelerated bone loss [1,6]. Beyond total adiposity, fat distribution is particularly important. The shift toward android (central) fat during menopause predicts cardiometabolic and musculoskeletal decline more strongly than overall body fat percentage (BF%), underscoring the relevance of measures such as the android-to-gynoid (A/G) fat ratio [7,8]. These changes increase the risk of obesity, sarcopenia, osteoporosis, frailty, and fractures [1‒6], conditions that disproportionately affect women. Osteoporosis is highly sex-skewed, affecting approximately one-third of women, who account for ∼80% of cases after age 40 [9]. Sarcopenia is similarly under-recognized, with prevalence estimates ranging from 10% to 40% in postmenopausal women depending on the definition applied [6]. Together, these outcomes contribute substantially to disability, health care use, and diminished quality of life among older women, highlighting midlife as a critical window for prevention and intervention [[10], [11], [12]].

Diet quality is 1 of the most important and modifiable influences on metabolic and musculoskeletal aging [3,13]. In this context, diet quality refers to the overall composition and patterning of foods consumed, reflecting both the nutritional value of foods and the extent of industrial processing [14,15]. Yet much of the existing literature has focused narrowly on individual nutrients. Although essential, individual nutrients do not capture the complexity of real-world eating patterns or the ways in which food processing and combinations of foods consumed across the day shape physiological responses [[14], [15], [16]]. Whole-diet approaches offer a more integrated perspective [14]. Mediterranean-style dietary patterns, rich in fruits, vegetables, whole grains, legumes, nuts, fish, and unsaturated fats [17,18], have been consistently linked to healthier adiposity profiles, improved metabolic regulation [17,19,20], and more favorable muscle [21] and bone indicators [22] in midlife and older women. Importantly, declines in lean mass during midlife exacerbate metabolic dysfunction [3] and increase the risk of sarcopenic obesity [12], underscoring the relevance of dietary quality for energy balance during aging.

At the same time, the modern food environment has shifted sharply toward industrially processed and ultraprocessed foods (UPFs) [15,23]. UPFs typically contain refined ingredients, additives, and cosmetic formulations that decrease nutrient density while increasing energy density and palatability [15,24,25]. Higher UPF consumption has been associated with greater central adiposity [26], poorer metabolic outcomes [27], lower muscle mass and strength [28], and lower bone mineral density (BMD) [29]. These patterns are particularly concerning for women, who experience physiological changes during midlife that may heighten vulnerability to diet-related metabolic and musculoskeletal decline [[2], [3], [4]].

Despite growing interest in diet quality and women’s aging, the evidence base remains fragmented, and women continue to be underrepresented in nutrition and musculoskeletal research. Existing studies typically examine Mediterranean-style diets and UPF consumption in separate bodies of work, rather than studying these contrasting dietary patterns within the same population. In addition, body composition and muscle function outcomes are usually investigated independently across distinct studies, limiting understanding of how diet quality relates to the multiple health domains that evolve during midlife. Finally, few investigations consider whether dietary associations differ before and after menopause, despite the marked hormonal and metabolic changes that accompany this transition. These gaps restrict our ability to understand how contemporary dietary patterns shape women’s metabolic, body composition, and functional health during a pivotal stage of aging.

To address these gaps, the present study evaluates how 2 contrasting whole-diet exposures, Mediterranean diet adherence and UPF consumption, relate to body composition and muscle function among midlife and older women in the United States. Using harmonized analytic methods across outcomes, we examine these associations within the same population and assess whether relationships differ according to menopausal status. This approach offers a comprehensive and policy-relevant perspective on the role of dietary quality in shaping key determinants of healthy aging in women.

## Methods

### Study design and population

The NHANES is a continuous, cross-sectional survey conducted by the National Center for Health Statistics (NCHS) using a complex, multistage probability sampling design to obtain a nationally representative sample of the civilian, noninstitutionalized United States population. Data collection includes household interviews, physical examinations, laboratory testing, and 2 standardized 24-h dietary recalls [30]. Body composition and muscle function measures are obtained in the mobile examination centers (MECs) following standardized NCHS protocols [31,32].

The analytic sample was determined by data availability within NHANES. Females aged ≥45 y from the 2007–2018 cycles were included if they completed both dietary recalls and had available data for BMI, dual-energy X-ray absorptiometry (DXA)–derived body composition measures, and/or grip strength assessments. Because NHANES examination and laboratory components vary by cycle, sample sizes differed across outcomes, and no a priori sample size calculation was performed.

Premenopausal females were included if they did not report being pregnant at the time of the examination. Information on breastfeeding status was not available in all of the NHANES analytic cycles included in this study and, therefore, could not be used as an exclusion criterion. Postmenopausal females were included if they reported natural menopause, defined as the absence of a menstrual period for ≥12 consecutive months, and provided complete information on the timing of their last menstrual period. Females were excluded if menopause occurred before age 40 or after age 62, or if amenorrhea was due to surgery (e.g., hysterectomy or oophorectomy). These criteria were applied to ensure the sample reflected typical physiological menopause and to minimize heterogeneity introduced by premature or medically induced menopausal states. Menopause before age 40 is consistent with primary ovarian insufficiency and is often accompanied by distinct etiologic and clinical factors, whereas menopause at older ages may reflect atypical reproductive aging trajectories. The upper age cutoff of 62 y was selected to align with prior population-based studies examining age at natural menopause and cardiometabolic and aging-related outcomes, thereby enhancing comparability with existing literature [33].

Participants missing required data for either dietary exposure or the outcome of interest were excluded from the relevant analysis. Covariates were handled using complete-case analysis within each model. Therefore, sample sizes declined as additional covariates were included and differ across models.

NHANES protocols were approved by the NCHS Research Ethics Review Board, and all participants provided written informed consent prior to participation. The present study used publicly available, de-identified data and was therefore exempt from additional institutional ethics approval.

### Dietary data and scoring

Dietary intake was assessed using two 24-h dietary recalls administered by trained interviewers using the USDA automated multiple-pass method, a validated protocol designed to improve recall accuracy [34]. Reported foods and beverages were linked to the USDA food and nutrient database for dietary studies for each NHANES cycle, and mean intake across the 2 recalls was used to represent usual intake [34].

Adherence to a Mediterranean-style diet was operationalized using a modified Mediterranean Diet Score (mMDS) [35]. Food-group intakes were derived from the USDA Food Patterns Equivalents Database, which groups NHANES food codes into cup- and ounce-equivalents [36]. The mMDS included the MUFAs to SFAs ratio, legumes/nuts/seeds, grains, fruits, vegetables and potatoes, fish, meat, and dairy [35]. We used a Mediterranean diet scoring system that excludes alcohol, consistent with current evidence indicating that even low levels of alcohol consumption confer health risks [37].

All food-group intakes were energy-adjusted to 2000 kcal/d for women using the density method, whereby each food-group intake was divided by total energy intake (kilocalories per day) and multiplied by 2000 [35]. Population-specific medians served as cut points, such that participants received 1 point if their intake was at or above the median and 0 points if below the median for each beneficial component, whereas lower intakes of meat and dairy (below the median) were scored favorably. Total scores ranged from 0 to 8, with higher values indicating greater adherence [35].

UPFs are industrial formulations typically made from refined substances and additives, with little or no intact whole foods, designed to be convenient, hyper-palatable, and ready-to-consume [15]. UPF intake was quantified using the NOVA classification system, which categorizes foods based on the extent and purpose of industrial processing, ranging from unprocessed or minimally processed foods (group 1), processed culinary ingredients (group 2), and processed foods (group 3), to UPFs (group 4). NOVA classifications were applied to NHANES food codes using an externally developed dataset based on previously published methods [24], and were not derived de novo in the present study. UPF consumption was expressed as the proportion of total grams per day derived from NOVA group 4 foods, calculated as grams of UPFs divided by total grams of all foods consumed. Although percent energy from UPFs is a commonly reported metric [38,39], a gram-based approach aligns more closely with NOVA’s processing-focused classification [15] and is appropriate in this context because it captures the relative volume of UPFs in the diet independent of their energy density, including lower–energy-density ultraprocessed items that contain cosmetic additives (e.g., nonsugar–sweetened beverages) [25,40]. This is an important consideration for body composition outcomes, as well as endocrine pathways that may be influenced by the quantity and formulation of UPFs rather than their caloric contribution [13,26,27].

Detailed food-group definitions, underlying food patterns equivalents database, and food and nutrient database for dietary studies variables, derivation procedures, energy-adjustment approach, and population-specific median cut points used to construct the mMDS, as well as the derivation of UPF variables, are provided in Supplementary Tables 1 and 2.

### Outcome variables

Outcomes of BMI, adiposity, muscle, and bone were measured. All outcomes were obtained from standardized NHANES examination components and DXA assessments conducted in the MEC.

BMI, measured in all analytic cycles, was derived from measured height and weight collected during the MEC physical examination. BMI was analyzed as a continuous variable and as a categorical variable using Centers for Disease Control and Prevention adult cutoffs: underweight (<18.5), healthy weight (18.5–24.9), overweight (25.0–29.9), and obesity (≥30.0) [41].

Total BF%, available for 2011–2018 cycles, was obtained from whole-body DXA scans conducted using Hologic systems [32]. A/G fat ratio, available for 2011–2018 cycles, was derived from DXA-measured fat mass in the android (abdominal) and gynoid (hip/thigh) regions. These regions are defined algorithmically by the Hologic DXA system based on standardized anatomical landmarks, with the android region reflecting central abdominal fat and the gynoid region encompassing the hips and upper thighs. Region-specific fat mass estimates are provided directly in the NHANES DXA datasets [32], and the derivation of these regions has been described in detail elsewhere [42].

Grip strength (kilogram), available for 2011–2014 cycles, was measured using a hand-held Takei dynamometer during the MEC examination. NHANES administered ≤3 trials per hand following standardized protocols [31]. The highest value from trials rated by the technician as “maximum effort” was retained for analysis.

ALM, available for 2011–2018 cycles, was calculated as the sum of appendicular lean soft tissue mass (kilogram) in both arms and legs obtained from DXA scans, excluding bone mineral content. ALM was normalized to BMI to derive the ALM/BMI index, consistent with validated sarcopenia definitions used in epidemiologic research [32,43].

Femoral-neck BMD (grams per square centimeter) was available in the 2007‒2010, 2013‒2014, and 2017‒2018 cycles, and was obtained from DXA scans performed in select NHANES cycles and provided as a derived variable in NHANES DXA datasets [32]. T-scores were calculated by standardizing femoral-neck BMD values relative to a young female adult reference mean (0.86 g/cm^2^) and SD (0.12 g/cm^2^) [44]. The T-score was used as the continuous BMD outcome.

Osteoporosis status was classified using femoral-neck T-score thresholds established by the Bone Health & Osteoporosis Foundation: healthy bone density (T-score ≥ –1.0), low bone density or osteopenia (–2.5 < T-score < –1.0), and osteoporosis (T-score ≤ –2.5) [45].

### Covariates

Covariates were selected a priori based on established associations with diet quality, body composition, and muscle function outcomes. Sociodemographic variables included age, race/ethnicity, education, and the family poverty-to-income ratio (PIR), using NHANES categories appropriate to each survey cycle. Lifestyle factors included mean daily energy intake, alcohol consumption (grams per day), smoking history (yes or no to smoking ≥100 cigarettes in lifetime), and meeting the United States aerobic physical activity guidelines of 150 min of moderate to vigorous physical activity [46].

Reproductive and clinical variables included menopausal status (pre- or postmenopausal) and menopausal hormone therapy (MHT) use, defined as ever use of estrogen- or progesterone-containing therapies excluding use for contraception or infertility. BMI was included as a covariate in models of grip strength, BMD, and osteoporosis due to its known physiological relationship with muscle and bone outcomes. Calcium (milligrams per day) and vitamin D (micrograms per day) supplement use were additionally included in BMD and osteoporosis models. Protein supplement use was not included as a covariate in muscle models because many protein supplements are classified as UPFs, and adjusting for their use could result in overadjustment or partial control of the primary exposure of interest. Chronic condition indicators were derived from self-reported physician diagnoses and included cardiovascular disease, type 2 diabetes, hypertension, hypercholesterolemia, and osteoarthritis.

### Statistical analyses

All analyses were conducted in SAS (version 9.4; SAS Institute Inc.) using survey procedures to account for NHANES’ complex, multistage probability sampling design, including appropriate strata and primary sampling units. Sample weights were selected in accordance with NHANES analytic guidelines based on the most restrictive component, defined as the smallest subsample containing all variables included in each model [47]. Variance estimation used Taylor series linearization.

Associations between dietary patterns and BMI, adiposity, muscle, and bone outcomes were evaluated using multivariable regression models. Dietary exposures were modeled as continuous variables to maximize statistical power and reflect potential dose-response relationships. Separate models were estimated for the mMDS and the proportion of UPFs consumed. Clinical outcomes were analyzed both continuously and categorically to facilitate interpretability and alignment with established clinical thresholds. For continuous outcomes, survey-weighted generalized linear models were used. For categorical outcomes, multinomial logistic regression was used to estimate associations with BMI and osteoporosis categories, using clinically meaningful reference groups (healthy weight and normal BMD) to facilitate interpretation.

Three levels of covariate adjustment were applied. Unadjusted models included only the dietary exposure and outcome to estimate crude associations. Minimally adjusted models (model 1) controlled for age, mean daily energy intake, race/ethnicity, education, and PIR quartile. Mean daily energy intake was included at this stage to account for differences in overall food consumption that may confound associations between dietary pattern measures and health outcomes, even when relative dietary indices were used. Fully adjusted models (model 2) additionally included smoking history, alcohol consumption, physical activity, menopausal status, MHT use, and chronic condition indicators (cardiovascular disease, type 2 diabetes, hypertension, hypercholesterolemia, and osteoarthritis). For grip strength, BMD, and osteoporosis outcomes, BMI was additionally included. For BMD and osteoporosis outcomes, vitamin D and calcium supplement use were also included. We did not mutually adjust Mediterranean diet adherence and UPF intake in the primary models because these indices are partially overlapping but capture related and distinct dimensions of diet quality, reflecting overall dietary composition and degree of food processing, respectively. Our analytic objective was to estimate the total association of each dietary exposure with health outcomes. Although these dietary dimensions may coexist within individuals, mutual adjustment or cross-classification would instead estimate conditional associations that isolate 1 exposure while holding the other constant. Such estimates may be more difficult to interpret in cross-sectional data and do not necessarily advance etiologic inference. Accordingly, our primary analyses focus on the overall associations of each dietary exposure, while acknowledging the potential for overlap.

Effect modification by menopausal status was evaluated using dietary exposure × menopausal status interaction terms, as this was a prespecified effect modifier. Additional interaction tests (e.g., age, race/ethnicity, smoking, and physical activity) were not pursued to limit multiple testing and maintain focus on primary objectives. As no statistically significant interactions were observed across outcomes, final models are presented without interaction terms. All tests were 2-sided with statistical significance defined as *P* < 0.05. Because outcomes represented distinct prespecified domains, no correction for multiple comparisons was applied. Model assumptions were assessed using standard diagnostics, including checks for multicollinearity, residual patterns, and overdispersion where applicable.

## Results

### Study population

The analytic sample included 8376 women aged 45 to 80 y. The weighted mean age was 61 ± 11 y, and the mean family PIR was 3.16 ± 1.64. Approximately one-third of participants had some college or an associate degree (31.92%), and a similar proportion were college graduates (28.86%). Most participants were non-Hispanic White (74.08%). Nearly half (45.50%) were postmenopausal, and approximately one-third reported ever using MHT (34.01%). Survey-weighted demographic, lifestyle, reproductive, and clinical characteristics are presented in Table 1.

**TABLE 1:** Survey-weighted demographic, lifestyle, reproductive, and clinical characteristics of women aged 45–80 y participating in NHANES, 2007–2018

Measure	Range	Weighted mean or %^1^	SD or 95% CI^1^
Age, y	45‒80	61	±11
Family PIR	0‒5.00	3.16	±1.64
Education level (%)			
<9th grade	—	5.26	4.54, 5.98
Some high school	—	9.75	8.72, 10.79
High school grad/GED/equivalent	—	24.15	22.76, 25.53
Some college/AA degree	—	31.92	30.14, 33.71
College grad or above	—	28.86	26.63, 31.09
Race/ethnicity (%)			
Mexican American	—	4.99	3.90, 6.09
Other Hispanic	—	4.47	3.64, 5.31
Non-Hispanic White	—	74.08	71.39, 76.77
Non-Hispanic Black	—	10.45	8.90, 11.99
Other/Multirace	—	6.01	5.11, 6.90
Menopausal status (%)			
Premenopausal	—	54.50	52.98, 56.02
Postmenopausal	—	45.50	43.98, 47.02
MHT use (%)			
Yes	—	34.01	32.59, 35.42
No	—	65.74	64.32, 67.16
Mean daily energy intake, kcal	156.00‒5679.00	1724.84	±588.12
Mean daily alcohol intake, g	0‒142.20	5.43	±13.35
Smoked ≥100 cigarettes (%)			
Yes	—	40.30	38.76, 41.84
No	—	59.65	58.13, 61.18
Meeting physical activity guidelines (%)			
Yes	—	49.82	48.13, 51.52
No	—	50.18	48.48, 51.87
Cardiovascular disease (%)			
Yes	—	11.98	10.99, 12.97
No	—	88.02	87.03, 89.01
Type 2 diabetes^2^ (%)			
Yes	—	13.86	12.74, 14.98
Borderline	—	2.98	2.52, 3.44
No	—	83.16	82.06, 84.27
Hypertension (%)			
Yes	—	47.48	45.93, 49.03
No	—	52.52	50.97, 54.07
Hypercholesterolemia (%)			
Yes	—	48.91	47.55, 50.27
No	—	51.09	49.73, 52.45
Osteoarthritis (%)			
Yes	—	21.45	19.98, 22.91
No	—	78.55	77.09, 80.02
Vitamin D supplement (μg/d)	0.004‒2570.00	37.85	±70.23
Calcium supplement (mg/d)	0.05‒4220.00	525.52	±515.94

Abbreviations: AA degree, Associate of Arts degree; CI, confidence interval; GED, General Educational Development; MHT, menopausal hormone therapy; PIR, poverty income ratio.

1 Values are survey-weighted means ± SDs for continuous variables and survey-weighted percentages (95% CI) for categorical variables.

2 Borderline type 2 diabetes reflects self-reported responses to physician diagnosis questions that included a “borderline” category.

### Exposures and outcomes

Survey-weighted distributions of dietary exposures and body composition, muscle, and bone outcomes are presented in Table 2. mMDS values spanned the full range of the scale, whereas UPF intake varied widely across participants. The sample was characterized by a high prevalence of overweight and obesity, along with elevated adiposity measures. Muscle outcomes, including ALM/BMI and grip strength, also demonstrated variability across the study population. Bone outcomes showed that most participants were classified as having normal bone density or osteopenia.

**TABLE 2:** Survey-weighted distributions of dietary exposures, body composition, and muscle function outcomes among women aged 45–80 y participating in NHANES 2007–2018

Measure	Range	Weighted mean or %^1^	SD or 95% CI^1^
mMDS (0‒8)	0‒8	3.80	±1.65
UPF consumption, proportion of daily food intake weight	0.001‒0.997	0.25	±0.17
BMI (kg/m^2^)	13.18‒84.40	30.18	±7.39
Underweight (%)	—	1.41	1.04, 1.78
Healthy weight (%)	—	27.17	25.74, 28.61
Overweight (%)	—	30.33	28.86, 31.80
Obese (%)	—	41.08	39.24, 42.93
Total body fat (% body weight)	18.50‒55.40	40.36	±5.54
A/G fat ratio	0.50‒1.60	0.94	±0.15
ALM (kg)/BMI (kg/m^2^)	0.33‒0.97	0.62	±0.10
Grip strength (kg)	6.10‒52.40	27.22	±6.17
Femoral-neck BMD (T-score)	‒5.97‒5.03	-1.02	±1.16
Osteoporosis classification			
Normal BMD (%)	—	44.49	42.66, 46.32
Osteopenia (%)	—	47.89	46.23, 49.55
Osteoporosis (%)	—	7.62	6.62, 8.62

Abbreviations: A/G, android-to-gynoid; ALM/BMI, appendicular lean mass normalized to body mass index; BMD, bone mineral density; CI, confidence interval; mMDS, modified Mediterranean Diet Score; UPF, ultraprocessed food.

1 Values are survey-weighted means ± SDs for continuous variables and survey-weighted percentages (95% CI) for categorical variables.

### BMI and adiposity outcomes

Higher mMDS was significantly associated with lower BMI and adiposity across all continuous measures. Each 1-point increase in mMDS was associated with significantly lower BMI, BF%, and A/G fat ratio (Table 3). Categorical models (Table 4) showed that higher mMDS was significantly associated with lower odds of overweight and obesity relative to healthy weight, with no significant association observed for underweight compared with healthy weight.

**TABLE 3:** Survey-weighted associations between dietary exposures and continuous measures of body composition and muscle function among women aged 45–80 y participating in NHANES, 2007–2018

	mMDS			NOVA UPF classification		
	*n*^5^	β^6^ (95% CI)	*P* value	*n*^5^	β^6^ (95% CI)	*P* value
BMI (kg/m^2^)						
Unadjusted	8376	–0.61 (–0.74, –0.47)	<0.0001	7930	5.78 (4.37, 7.19)	<0.0001
Model 1^1^	7144	–0.51 (–0.66, –0.37)	<0.0001	7140	4.32 (2.79, 5.86)	<0.0001
Model 2^2^	6452	–0.37 (–0.51, –0.24)	<0.0001	6448	2.97 (1.52, 4.42)	0.0001
Total body fat (%)						
Unadjusted	1920	‒0.64 (–0.87, –0.41)	<0.0001	1771	6.04 (3.57, 8.50)	<0.0001
Model 1^1^	1620	‒0.61 (–0.87, –0.35)	<0.0001	1618	5.57 (2.84, 8.30)	0.0002
Model 2^2^	1527	‒0.46 (–0.72, –0.20)	0.0009	1525	4.72 (2.39, 7.05)	0.0002
A/G fat ratio						
Unadjusted	2114	‒0.016 (–0.022, –0.010)	<0.0001	1950	0.06 (0.01, 0.12)	0.0385
Model 1^1^	1780	‒0.016 (–0.023, –0.009)	<0.0001	1779	0.053 (−0.005, 0.111)	0.0704
Model 2^2^	1676	‒0.014 (–0.021, –0.007)	<0.0001	1675	0.060 (0.001, 0.120)	0.0455
ALM (kg)/BMI (kg/m^2^)						
Unadjusted	1904	0.009 (0.005, 0.012)	<0.0001	1753	‒0.06 (–0.10, –0.02)	0.0092
Model 1^1^	1607	0.009 (0.005, 0.012)	<0.0001	1605	‒0.09 (–0.12, –0.05)	<0.0001
Model 2^2^	1516	0.006 (0.003, 0.010)	0.0011	1514	‒0.07 (–0.11, –0.04)	0.0001
Grip strength (kg)						
Unadjusted	2499	0.01 (–0.20, 0.22)	0.9243	2415	0.80 (–0.85, 2.45)	0.3509
Model 1^1^	2222	0.07 (–0.11, 0.25)	0.4442	2219	‒2.02 (–3.58, –0.45)	0.0166
Model 2^2^^,^^3^	2094	0.08 (–0.10, 0.27)	0.3676	2091	‒1.88 (–3.64, –0.11)	0.0449
BMD (T-score)						
Unadjusted	4601	−0.005 (–0.031, 0.022)	0.7366	4429	0.34 (0.12, 0.56)	0.0037
Model 1^1^	4005	0.005 (–0.024, 0.033)	0.7291	4004	0.04 (–0.24, 0.33)	0.7572
Model 2^2^, ^3^, ^4^	1648	0.029 (-0.001, 0.060)	0.0556	1648	0.06 (–0.24, 0.36)	0.6980

Abbreviations: A/G, android-to-gynoid; ALM/BMI, appendicular lean mass normalized to body mass index; BMD, bone mineral density; CI, confidence interval; DXA: dual-energy X-ray absorptiometry; mMDS, modified Mediterranean Diet Score; UPF, ultraprocessed food.

1 Model 1 adjusted for age, mean daily energy intake, race/ethnicity, education, and family poverty–income ratio.

2 Model 2 additionally adjusted for smoking history, alcohol consumption, physical activity, menopausal status, menopausal hormone therapy use, and chronic condition indicators (cardiovascular disease, type 2 diabetes, hypertension, hypercholesterolemia, and osteoarthritis).

3 For grip strength and BMD outcomes, BMI was additionally included.

4 For BMD outcome, vitamin D and calcium supplement use were also included.

5 Sample sizes vary across outcomes and models due to the availability of DXA measures and covariate-specific missing data.

6 β coefficients represent the change in outcome per 1-unit increase in the mMDS or per 1-unit increase in the proportion of dietary intake from UPFs.

**TABLE 4:** Survey-weighted associations between dietary exposures and categorical classifications of BMI and osteoporosis among women aged 45–80 y participating in NHANES 2007–2018

	mMDS			NOVA UPF classification		
	*n*^4^	Odds ratio^5^ (95% CI)	*P* value	*n*^4^	Odds ratio^5^ (95% CI)	*P* value
BMI						
Unadjusted	8376	—		7930	—	
Underweight	—	0.93 (0.78, 1.13)	0.4680	—	7.52 (0.99, 57.35)	0.0517
Healthy weight	—	Reference		—	Reference	
Overweight	—	0.92 (0.88, 0.96)	<0.0001	—	1.75 (1.02, 3.01)	0.0417
Obese	—	0.83 (0.79, 0.87)	<0.0001	—	5.55 (3.23, 9.55)	<.0001
Model 1^1^	7144	—		7140	—	
Underweight	—	1.01 (0.80, 1.27)	0.9309	—	5.93 (0.88, 40.23)	0.0678
Healthy weight	—	Reference		—	Reference	
Overweight	—	0.93 (0.89, 0.98)	0.0047	—	1.57 (0.88, 2.83)	0.1286
Obese	—	0.85 (0.80, 0.89)	<0.0001	—	4.10 (2.25, 7.46)	<.0001
Model 2^2^	6452	—		6448	—	
Underweight	—	0.96 (0.77, 1.20)	0.7205	—	7.22 (0.85, 61.22)	0.0700
Healthy weight	—	Reference		—	Reference	
Overweight	—	0.94 (0.90, 0.99)	0.0168	—	1.45 (0.82, 2.56)	0.2002
Obese	—	0.87 (0.82, 0.93)	<0.0001	—	2.96 (1.61, 5.44)	0.0005
Osteoporosis						
Unadjusted	4601	—		4429	—	
Healthy BMD	—	Reference		—	Reference	
Osteopenia	—	1.03 (0.98, 1.08)	0.1839	—	0.45 (0.25, 0.80)	0.0077
Osteoporosis	—	0.91 (0.83, 1.00)	0.0487	—	1.15 (0.46, 2.89)	0.7595
Model 1^1^	4005	—		4004	—	
Healthy BMD	—	Reference		—	Reference	
Osteopenia	—	1.03 (0.96, 1.09)	0.4263	—	0.71 (0.37, 1.36)	0.2985
Osteoporosis	—	0.86 (0.76, 0.97)	0.0164	—	3.24 (1.12, 9.41)	0.0311
Model 2^2^^,^^3^	1648	—		1648	—	
Healthy BMD	—	Reference		—	Reference	
Osteopenia	—	0.91 (0.84, 0.98)	0.0188	—	1.23 (0.50, 3.01)	0.6530
Osteoporosis	—	0.82 (0.66, 1.01)	0.0642	—	1.25 (0.22, 7.23)	0.8025

Abbreviations: BMD, bone mineral density; CI, confidence interval; mMDS, modified Mediterranean Diet Score; UPF, ultraprocessed food.

1 Model 1 adjusted for age, mean daily energy intake, race/ethnicity, education, and family poverty–income ratio.

2 Model 2 additionally adjusted for smoking history, alcohol consumption, physical activity, menopausal status, menopausal hormone therapy use, and chronic condition indicators (cardiovascular disease, type 2 diabetes, hypertension, hypercholesterolemia, and osteoarthritis).

3 For bone outcomes, Model 2 was further adjusted for BMI, and calcium and vitamin D supplement use.

4 Sample sizes vary across outcomes and models due to availability of DXA measures and covariate-specific missing data.

5 Odds ratios represent the relative odds of being in each category compared with the reference group per 1-unit increase in the mMDS or per 1-unit increase in the proportion of dietary intake from UPFs.

Higher UPF consumption revealed inverse relationships. Across models, greater UPF intake was significantly associated with higher BMI, BF%, and a higher A/G fat ratio in the unadjusted and fully adjusted (model 2) models, but not in the minimally adjusted (model 1) model. In categorical analyses, higher UPF intake was associated with greater odds of obesity relative to healthy weight across all models, and with greater odds of overweight only in the unadjusted model. There was no significant difference in UPF intake for underweight relative to healthy weight.

### Muscle outcomes

Higher mMDS was significantly associated with greater ALM/BMI across models, indicating more favorable muscle composition, but was not associated with grip strength in any model. Higher UPF intake was significantly inversely associated with ALM/BMI and grip strength in model 1 and model 2.

### Bone outcomes

mMDS was not significantly associated with continuous femoral-neck BMD. In categorical analyses, higher mMDS was significantly associated with lower odds of having osteoporosis in the unadjusted model and model 1, and with lower odds of osteopenia only in model 2. For UPF intake, significant associations with continuous BMD were present only in the unadjusted model and were not significant after adjustment. In categorical models, higher UPF intake was associated with significantly lower odds of osteopenia in the unadjusted model and greater odds of osteoporosis in model 1. Neither association persisted after full adjustment.

### Effect modification by menopausal status

Menopausal status did not significantly modify the associations between dietary exposure and any outcome of interest. Interaction terms between menopausal status and both mMDS and UPF intake were not statistically significant across all outcomes (all interaction *P* values >0.05), indicating comparable associations in premenopausal and postmenopausal females.

## Discussion

In this nationally representative sample of United States women aged 45 to 80 y, higher adherence to a Mediterranean-style dietary pattern was associated with lower BMI and adiposity, and more favorable relative muscle mass, whereas higher UPF consumption was associated with greater BMI and adiposity, and less favorable muscle composition. Associations between UPF consumption and grip strength were present but more modest, and neither dietary pattern showed robust associations with bone outcomes after full adjustment. Importantly, these relationships were consistent across menopausal status. Together, these findings indicate that overall diet quality, capturing both protective and detrimental patterns, is strongly related to BMI, adiposity, and muscle mass and function during midlife and older adulthood in women, whereas associations with bone appear weaker in cross-sectional data.

Higher adherence to a Mediterranean-style dietary pattern may influence adiposity and muscle health through several interrelated biological mechanisms that are particularly relevant during midlife and postmenopause. Consistent with the lower adiposity and more favorable muscle mass relative to body size observed in this study, Mediterranean dietary patterns are characterized by higher intakes of plant-based foods, fish, unsaturated fats, and other nutrient-dense foods rich in fiber, antioxidants, and essential micronutrients [17]. These features have been associated with lower systemic inflammation and improved insulin sensitivity [17,48]. In particular, higher-quality protein intake, micronutrient density (e.g., magnesium, potassium), and anti-inflammatory lipid profiles, hallmarks of Mediterranean-style dietary patterns, have been associated with higher muscle mass, improved muscle strength, and lower prevalence of sarcopenia, and may support skeletal muscle maintenance while contributing to more favorable fat distribution and central adiposity, particularly during periods of hormonal transition [22,49,50].

In contrast, the greater adiposity and less favorable muscle composition associated with higher UPF consumption in our analyses may reflect differences in dietary composition and metabolic quality. These include higher glycemic load and refined carbohydrate content, a higher proportion of saturated fats and lower intake of unsaturated fats, potential exposure to food additives, contact materials, and endocrine-disrupting chemicals arising from processing and packaging, and displacement of protein and micronutrients critical for muscle and skeletal tissues [15,25,40,51,52]. Together with mechanisms that may promote increased energy intake, these factors may contribute to adverse body composition profiles. During menopause, when estrogen-related protection against fat accumulation and muscle loss diminishes, these dietary characteristics may exacerbate unfavorable body composition phenotypes, consistent with the associations observed in this cohort [2,3,26,38].

This study focuses specifically on women during midlife and older adulthood, a period marked by substantial hormonal and physiological changes that have important implications for body composition and metabolic health. Midlife reproductive aging and the postmenopausal period are associated with shifts toward increased fat mass, central adiposity, and loss of muscle mass, processes that may interact with dietary exposures in sex-specific ways due to underlying hormonal and metabolic differences [2,3,13]. Despite the importance of the menopausal transition for body composition, women remain underrepresented in nutrition and aging research, and many studies extrapolate findings from mixed-sex or male-dominated samples. By examining dietary patterns in relation to DXA-derived measures of adiposity and musculoskeletal outcomes among women, this study provides sex-specific evidence relevant to understanding obesity phenotypes and muscle and bone health during aging.

To our knowledge, this study is among the first to evaluate protective dietary patterns and UPF consumption in parallel, using objective anthropometric and DXA-derived measures of multiple aspects of body composition, within a nationally representative cohort of midlife and older women. Most prior research has examined single dietary constructs, focused on nutrients rather than food processing, or combined men and women across wide age ranges. By explicitly targeting women across midlife and older adulthood, accounting for menopausal status, and by distinguishing between BMI, adiposity, muscle, and bone outcomes, this study provides domain-specific insight into how diet quality relates to obesity-relevant body composition changes during aging. By operationalizing UPF intake as a processing-based exposure independent of energy density, this study contributes to the emerging literature emphasizing food processing, not just nutrient content, as a determinant of obesity-related outcomes in aging populations [15,40].

Adipose tissue and skeletal muscle are metabolically dynamic and respond relatively quickly to changes in dietary composition, energy balance, and food processing [2,3]. In contrast, bone density reflects cumulative exposures over decades and is strongly influenced by long-term mechanical loading, hormonal history, and mineral homeostasis [22,53]. The present findings align with this biological distinction, suggesting that diet quality in midlife may exert more detectable effects on soft tissue compartments than on skeletal outcomes within cross-sectional analyses.

Higher Mediterranean diet adherence was associated with lower BMI, lower total and central adiposity, and higher ALM relative to body size. These findings are consistent with prior evidence linking Mediterranean-style eating to healthier fat distribution [49] and cardiometabolic profiles [17] and extend this literature by demonstrating concordant associations across multiple DXA-derived adiposity measures. The association with relative lean mass is particularly relevant in the context of obesity during aging, as preservation of muscle mass mitigates metabolic dysfunction and reduces risk of sarcopenic obesity [12].

Conversely, higher UPF consumption was associated with greater BMI and adiposity, a more adverse android fat distribution, and lower relative lean mass, all relative to lower UPF consumption. These findings align with growing evidence that ultraprocessed diets contribute to positive energy balance through high energy density, low satiety potential, and displacement of nutrient-dense foods [15,25,52]. Although grip strength was not significantly associated with the mMDS, it was inversely associated with UPF intake in adjusted models, suggesting potential implications for muscle function as well as composition. Grip strength is not only a marker of muscle function but also a well-established predictor of future functional limitations and disability, as well as all-cause and cardiovascular mortality in midlife and older adults [[54], [55], [56]]. As such, the observed inverse association between UPF intake and grip strength may have implications that extend beyond muscle performance alone. If confirmed in longitudinal studies, these findings raise the possibility that high UPF intake may contribute to downstream functional decline and adverse health outcomes through pathways distinct from overall diet quality.

In contrast to BMI, adiposity, and muscle measures, which showed significant associations with the dietary exposures, associations between dietary exposures and bone measures were weak and inconsistent, with no robust associations observed for femoral-neck bone density or osteoporosis classification after full adjustment. These null findings likely reflect both biological and methodological factors. Bone density changes gradually and may be less responsive to short-term dietary variation captured by 24-h recalls, whereas the relatively low prevalence of osteoporosis and restriction of DXA measures to selected NHANES cycles reduced statistical power for categorical outcomes. Collectively, these findings suggest that diet quality may influence BMI, adiposity, and muscle outcomes more consistently than bone health in midlife women, and that cross-sectional designs may be less sensitive for detecting diet-bone associations.

Despite substantial hormonal and metabolic changes associated with menopause and midlife reproductive aging, we did not observe evidence of effect modification by menopausal status. This suggests that associations between diet quality and BMI, adiposity, or muscle outcomes operate similarly across midlife and older adulthood. Nonetheless, these findings indicate that dietary quality remains relevant both before and after menopause, supporting the potential value of dietary interventions throughout midlife rather than exclusively in the postmenopausal period.

Given that the study population included women aged ≥45 y, a substantial proportion of participants were likely in the menopausal transition. This age restriction was applied to focus on midlife and older women, in whom menopausal transition and postmenopausal physiology are most relevant to the study objectives, and to reduce heterogeneity introduced by including younger premenopausal women with substantially different hormonal profiles and body composition trajectories. However, menopausal status in this study was classified dichotomously (pre- compared with postmenopausal), which does not capture the continuum of reproductive aging. This approach may lead to misclassification of women in the menopausal transition, a period characterized by rapid hormonal and physiological changes that may differentially influence body composition and metabolic responses. Greater granularity, such as stratification by stages of the menopausal transition defined by established frameworks like the Stages of Reproductive Aging Workshop criteria [57], may have revealed heterogeneity across perimenopausal and postmenopausal sub-stages that could not be assessed here with the available data. Thus, the absence of observed effect modification may partly reflect the limited resolution of menopausal status rather than true uniformity of associations.

Beyond menopausal status, further investigation of the joint effects of overall diet quality and food processing may yield additional insight into obesity-related body composition outcomes. Although Mediterranean diet adherence and UPF consumption were examined independently in the present study, these dietary dimensions are not mutually exclusive and may coexist within individuals, reflecting overlapping but distinct aspects of diet quality. Although higher adherence to a Mediterranean-style pattern may correspond with lower intake of UPF, this relationship is not deterministic [58]. In this context, our analytic approach focused on estimating the total association of each dietary exposure rather than attempting to partition its shared variance. Approaches that mutually adjust for both indices or examine cross-classified exposure groups would estimate conditional or joint effects that are conceptually distinct and may be less interpretable in cross-sectional data. Prior work suggests that high adherence to a Mediterranean-style diet may not fully offset the adverse health effects associated with high UPF intake, and conversely, which lower UPF consumption may confer benefits even among individuals with lower adherence to traditional dietary patterns [58]. Future studies could extend this work by examining combined exposure categories (e.g., high compared with low Mediterranean diet adherence crossed with high compared with low UPF intake) or formally assessing additive and multiplicative interactions, which may help disentangle the relative and joint contributions of dietary pattern quality and food processing to body composition and functional aging in women.

The inclusion of DXA-derived measures represents a key methodological strength that meaningfully extends prior work in this area. Most existing studies examining dietary patterns in relation to obesity or aging-related outcomes rely primarily on BMI or waist circumference, which do not distinguish between fat mass, lean mass, or fat distribution [[59], [60], [61]]. By incorporating DXA-derived assessments of total and regional adiposity, appendicular lean soft tissue mass, and femoral-neck bone density, this study provides a more precise characterization of body composition and allows domain-specific evaluation of BMI, adiposity, muscle, and skeletal components. This level of phenotyping enables detection of associations that may be obscured when using cruder anthropometric measures alone and is particularly relevant for midlife and older women, in whom changes in fat distribution and muscle mass may occur independently of overall body weight. Importantly, the use of DXA-based outcomes also facilitates clearer interpretation of observed associations, distinguishing diet-related differences in soft tissue composition from skeletal measures that reflect longer-term cumulative exposures.

However, several limitations inherent to the design and conduct of this study should be considered when interpreting these findings. The cross-sectional design precludes causal inference and raises the possibility of reverse causation, whereby women who have obesity or chronic conditions may have modified dietary patterns prior to assessment. Such behavior change would be expected to attenuate observed associations, potentially biasing estimates toward the null. Dietary intake was assessed under free-living conditions in a nationally representative, noninstitutionalized United States population, which reflects usual dietary behaviors but relies on self-reported intake and may be influenced by the broader food environment. In addition, dietary intake was assessed using two 24-h recalls, which are not specifically designed to capture food processing and therefore may result in nondifferential misclassification of UPF intake, further attenuating associations. Systematic underreporting of UPFs among individuals with higher adiposity could also bias associations toward the null. Finally, dietary recalls remain subject to recall error and may not fully capture long-term dietary patterns relevant to body composition and musculoskeletal aging.

Information on reproductive history beyond menopausal status was not incorporated into analytic models. However, in this cohort of women aged 45 to 80 y, postpartum-related changes in body composition are unlikely to persist decades after childbirth, and adjustment for age is expected to capture the dominant time-related influences on adiposity and muscle outcomes. In addition, DXA-derived measures were available only in selected NHANES cycles and are subject to eligibility restrictions and nonresponse, reducing sample size and potentially introducing selection bias for bone and body composition analyses. MHT use was assessed as ever use, without information on duration, timing, formulation, or cumulative exposure. As a result, women who reported MHT use may have been exposed for markedly different periods, ranging from short-term use to many years of therapy, which likely have very different implications for body composition and musculoskeletal outcomes. Accordingly, no conclusions can be drawn regarding the potential modifying or independent effects of MHT on the associations observed in this study. Medication use was captured as a binary (yes/no) self-reported variable indicating use in the past 30 d. Although medications were coded using standardized drug classifications, this measure does not capture duration of use, adherence, or indication. Moreover, uptake of newer antiobesity pharmacotherapies and some osteoporosis treatments was low during much of the 2007–2018 period, limiting our ability to account for their potential influence. Nonetheless, the consistency of associations observed across multiple BMI, adiposity, and muscle measures strengthens confidence in the overall pattern of findings.

The findings of this study are broadly generalizable to midlife and older women in the United States, as NHANES is designed to be nationally representative of the noninstitutionalized United States population. Use of survey weights enhances external validity across diverse racial/ethnic and socioeconomic groups. However, results may not generalize to men, institutionalized populations, women outside the 45 to 80 y age range, or populations with substantially different dietary patterns or food environments. Caution is also warranted when extrapolating these findings to settings with lower availability or consumption of UPFs.

In midlife and older United States women, higher adherence to a Mediterranean-style diet and lower consumption of UPFs are associated with more favorable BMI, adiposity, and muscle profiles, whereas associations with bone outcomes are less evident in cross-sectional data. These findings highlight the importance of examining BMI, adiposity, muscle, and bone as related but distinct domains and underscore the relevance of food processing and overall dietary quality for understanding obesity during aging. In the context of recent dietary guideline updates emphasizing whole dietary patterns and reduced intake of UPFs, these findings suggest that greater adherence to such recommendations could contribute to more favorable body composition outcomes in midlife and older women [62]. Longitudinal and intervention studies are needed to clarify causal pathways and to determine whether sustained improvements in diet quality translate into long-term benefits for body composition and functional outcomes during aging in women.

## Author contributions

The authors’ responsibilities were as follows– BS, JH, KB, AS, AC: designed the research; BS: conducted the research; EM-S: provided essential materials and expertise related to ultraprocessed food classification; BS: analyzed the data; BS, AC: wrote the paper, and had primary responsibility for final content; AB: contributed to study development and provided conceptual input; and all authors: read and approved the final manuscript.

## Data availability

Data described in the manuscript, code book, and analytic code will be made available upon request, pending application and approval.

## Declaration of Generative AI and AI-Assisted Technologies in the Writing Process

The authors declare that no generative AI or AI-assisted technologies were used in the writing of this manuscript.

## Funding

The authors reported no funding received for this study.

## Conflict of interest

The authors report no conflicts of interest.

## References

[1] Santoro N. (2005). The menopausal transition. Am. J. Med..

[2] Greendale G.A., Sternfeld B., Huang M., Han W., Karvonen-Gutierrez C., Ruppert K. (2019). Changes in body composition and weight during the menopause transition. JCI Insight..

[3] Pataky M.W., Young W.F., Nair K.S. (2021). Hormonal and metabolic changes of aging and the influence of lifestyle modifications. Mayo Clin. Proc..

[4] Mauvais-Jarvis F., Lindsey S.H. (2024). Metabolic benefits afforded by estradiol and testosterone in both sexes: clinical considerations. J. Clin. Invest..

[5] Horstman A.M., Dillon E.L., Urban R.J., Sheffield-Moore M. (2012). The role of androgens and estrogens on healthy aging and longevity, J. Gerontol. A Biol. Sci. Med. Sci..

[6] Sirola J., Kröger H. (2011). Similarities in acquired factors related to postmenopausal osteoporosis and sarcopenia. J. Osteoporos..

[7] Okosun I.S., Seale J.P., Lyn R. (2015). Commingling effect of gynoid and android fat patterns on cardiometabolic dysregulation in normal weight American adults. Nutr. Diabetes.

[8] Nistor I.M., Fica S., Martin S.C., Mustata T., Oprea T.E., Sirbu A.E. (2024). DXA android-to-gynoid ratio and cardiovascular risk assessment in age and BMI propensity-matched early postmenopausal women. Medicina (Kaunas).

[9] Osteoporosis Canada. Facts and stats [Internet]. Osteoporosis Canada; [cited 19 December, 2025]. Available from: https://osteoporosis.ca/facts-and-stats/.

[10] Crocker T.F., Brown L., Clegg A., Farley K., Franklin M., Simpkins S. (2019). Quality of life is substantially worse for community-dwelling older people living with frailty: systematic review and meta-analysis. Qual. Life Res..

[11] Bandeen-Roche K., Seplaki C.L., Huang J., Buta B., Kalyani R.R., Varadhan R. (2015). Frailty in older adults: a nationally representative profile in the United States. J. Gerontol. A Biol. Sci. Med. Sci..

[12] Batsis J.A., Mackenzie T.A., Lopez-Jimenez F., Bartels S.J. (2015). Sarcopenia, sarcopenic obesity, and functional impairments in older adults: national Health and Nutrition Examination Surveys 1999-2004. Nutr. Res..

[13] Feskens E.J., Bailey R., Bhutta Z., Biesalski H.K., Eicher-Miller H., Krämer K. (2022). Women’s health: optimal nutrition throughout the lifecycle. Eur. J. Nutr..

[14] Hu F.B. (2002). Dietary pattern analysis: a new direction in nutritional epidemiology. Curr. Opin. Lipidol..

[15] Monteiro C.A., Cannon G., Levy R.B., Moubarac J.C., Louzada M.L., Rauber F. (2019). Ultra-processed foods: what they are and how to identify them. Public Health Nutr.

[16] Jacobs D.R., Gross M.D., Tapsell L.C. (2009). Food synergy: an operational concept for understanding nutrition. Am. J. Clin. Nutr..

[17] Martínez-González M.A., Gea A., Ruiz-Canela M. (2019). The Mediterranean diet and cardiovascular health. Circ. Res..

[18] Trichopoulou A., Costacou T., Bamia C., Trichopoulos D. (2003). Adherence to a Mediterranean diet and survival in a Greek population. N. Engl. J. Med..

[19] Salas-Salvadó J., Bulló M., Babio N., Martínez-González M.Á., Ibarrola-Jurado N., Basora J. (2011). Reduction in the incidence of type 2 diabetes with the Mediterranean diet: results of the PREDIMED-Reus nutrition intervention randomized trial. Diabetes Care.

[20] Pugliese G., Barrea L., Laudisio D., Aprano S., Castellucci B., Framondi L. (2020). Mediterranean diet as tool to manage obesity in menopause: a narrative review. Nutrition.

[21] Mohseni R., Aliakbar S., Abdollahi A., Yekaninejad M.S., Maghbooli Z., Mirzaei K. (2017). Relationship between major dietary patterns and sarcopenia among menopausal women. Aging Clin. Exp. Res..

[22] Rizzoli R., Biver E., Brennan-Speranza T.C. (2021). Nutritional intake and bone health. Lancet Diabetes Endocrinol.

[23] Juul F., Parekh N., Martinez-Steele E., Monteiro C.A., Chang V.W. (2022). Ultra-processed food consumption among US adults from 2001 to 2018. Am. J. Clin. Nutr..

[24] Steele E.M., O’Connor L.E., Juul F., Khandpur N., Galastri Baraldi L.G., Monteiro C.A. (2023). Identifying and estimating ultraprocessed food intake in the US NHANES according to the Nova classification system of food processing. J. Nutr..

[25] Lane M.M., Gamage E., Du S., Ashtree D.N., McGuinness A.J., Gauci S. (2024). Ultra-processed food exposure and adverse health outcomes: umbrella review of epidemiological meta-analyses. BMJ.

[26] Liu J., Steele E.M., Li Y., Yi S.S., Monteiro C.A., Mozaffarian D. (2023). Consumption of ultraprocessed foods and body fat distribution among U.S. adults. Am. J. Prev. Med..

[27] Grinshpan L.S., Eilat-Adar S., Ivancovsky-Wajcman D., Kariv R., Gillon-Keren M., Zelber-Sagi S. (2023). Ultra-processed food consumption and non-alcoholic fatty liver disease, metabolic syndrome and insulin resistance: a systematic review. JHEP Rep.

[28] Almeida H.C., Lage V.K., Paula F.A., Freitas J.P., Moreno L.G., Oliveira V.C. (2022). Association between ultra-processed foods frequency of intake and sarcopenia in older adults: a cross-sectional study. Res. Soc. Dev..

[29] Ciaffi J., Mancarella L., Ripamonti C., D’Amuri A., Brusi V., Pignatti F. (2025). Ultra-processed food and its impact on bone health and joint diseases: a scoping review. Nutrients.

[30] CDC NHANES About page. About NHANES [Internet]. US Centers for Disease Control and Prevention; [cited 19 December, 2025]. Available from: https://www.cdc.gov/nchs/nhanes/about/index.html..

[31] CDC NHANES Muscle Strength Procedures Manual (2013). https://wwwn.cdc.gov/nchs/data/nhanes/public/2013/manuals/Muscle_Strength_2013.pdf.

[32] CDC NHANES Body Composition Procedures Manual (2018). https://wwwn.cdc.gov/nchs/data/nhanes/public/2017/manuals/Body_Composition_Procedures_Manual_2018.pdf.

[33] Appiah D., Nwabuo C.C., Ebong I.A., Wellons M.F., Winters S.J. (2021). Trends in age at natural menopause and reproductive life span among US women, 1959-2018. JAMA.

[34] Ahluwalia N., Dwyer J., Terry A., Moshfegh A., Johnson C. (2016). Update on NHANES dietary data: focus on collection, release, analytical considerations, and uses to inform public policy. Adv. Nutr..

[35] Knoops K.T., de Groot L.C., Kromhout D., Perrin A.E., Moreiras-Varela O., Menotti A. (2004). Mediterranean diet, lifestyle factors, and 10-year mortality in elderly European men and women: the HALE project. JAMA.

[36] Waller A.W., Martin C.L., Morton S., Friday J.E., Myrowitz R., Moshfegh A.J. (2025). Food Patterns Equivalents Database: development parameters and implications for the future. J. Food Compos. Anal..

[37] Anderson B.O., Berdzuli N., Ilbawi A., Kestel D., Kluge H.P., Krech R. (2023). Health and cancer risks associated with low levels of alcohol consumption. Lancet Public Health.

[38] Juul F., Martinez-Steele E., Parekh N., Monteiro C.A., Chang V.W. (2018). Ultra-processed food consumption and excess weight among US adults. Br. J. Nutr..

[39] Wang L., Martínez Steele E., Du M., Pomeranz J.L., O’Connor L.E., Herrick K.A. (2021). Trends in consumption of ultraprocessed foods among US youths aged 2-19 years, 1999-2018. JAMA.

[40] Dicken S.J., Batterham R.L. (2021). The role of diet quality in mediating the association between ultra-processed food intake, obesity and health-related outcomes: a review of prospective cohort studies. Nutrients.

[41] CDC Adult BMI Categories. Adult BMI categories [Internet]. Centers for Disease Control and Prevention; [cited 19 December, 2026]. Available from: https://www.cdc.gov/bmi/adult-calculator/bmi-categories.html.

[42] Bell K.E., Paris M.T., Avrutin E., Mourtzakis M. (2022). Altered features of body composition in older adults with type 2 diabetes and prediabetes compared with matched controls. J. Cachexia. Sarcopenia. Muscle..

[43] Studenski SA, Peters KW, Alley DE, Cawthon PM, McLean RR, Harris TB (2014). The FNIH sarcopenia project: rationale, study description, conference recommendations, and final estimates. J. Gerontol. A Biol. Sci. Med. Sci..

[44] Looker A.C., Orwoll E.S., Johnston C.C., Lindsay R.L., Wahner H.W., Dunn W.L. (1997). Prevalence of low femoral bone density in older U.S. adults from NHANES III. J. Bone Miner. Res..

[45] Bone density test, osteoporosis screening & T-score interpretation [Internet]. Osteoporosis Canada; [cited 19 December, 2025]. Available from: https://www.bonehealthandosteoporosis.org/patients/diagnosis-information/bone-density-examtesting/.

[46] Piercy K.L., Troiano R.P., Ballard R.M., Carlson S.A., Fulton J.E., Galuska D.A. (2018). The physical activity guidelines for Americans. JAMA.

[47] CDC NHANES Analytic Guidelines and Estimation Procedures. National health and nutrition examination survey, 2015-2018: sample design and estimation procedures [Internet]. US Centers for Disease Control and Prevention; [cited 19 December, 2025]. Available from: https://wwwn.cdc.gov/nchs/nhanes/analyticguidelines.aspx#estimation-and-weighting-procedures..

[48] Vetrani C., Verde L., Colao A., Barrea L., Muscogiuri G. (2023). The Mediterranean diet: effects on insulin resistance and secretion in individuals with overweight or obesity. Nutrients.

[49] Álvarez-Pérez J., Sánchez-Villegas A., Díaz-Benítez E.M., Ruano-Rodríguez C., Corella D., Martínez-González M.Á. (2016). Influence of a Mediterranean dietary pattern on body fat distribution: results of the PREDIMED-Canarias intervention randomized trial. J. Am. Coll. Nutr..

[50] Mazza E., Ferro Y., Maurotti S., Micale F., Boragina G., Russo R. (2024). Association of dietary patterns with sarcopenia in adults aged 50 years and older. Eur. J. Nutr..

[51] Srour B., Fezeu L.K., Kesse-Guyot E., Allès B., Méjean C., Andrianasolo R.M. (2019). Ultra-processed food intake and risk of cardiovascular disease: prospective cohort study (NutriNet-Santé). BMJ.

[52] Hall K.D., Ayuketah A., Brychta R., Cai H., Cassimatis T., Chen K.Y. (2020). Ultra-processed diets cause excess calorie intake and weight gain: an inpatient randomized controlled trial of ad libitum food intake. Cell Metab..

[53] Weaver C.M., Gordon C.M., Janz K.F., Kalkwarf H.J., Lappe J.M., Lewis R. (2016). The National Osteoporosis Foundation’s position statement on peak bone mass development and lifestyle factors: a systematic review and implementation recommendations. Osteoporos. Int..

[54] Leong D.P., Teo K.K., Rangarajan S., Lopez-Jaramillo P., Avezum A., Orlandini A. (2015). Prognostic value of grip strength: findings from the Prospective Urban Rural Epidemiology (PURE) study. Lancet..

[55] Bohannon R.W. (2019). Grip strength: an indispensable biomarker for older adults. Clin. Interv. Aging..

[56] Cooper R., Kuh D., Hardy R. (2010). Mortality Review Group, Falcon and HALCyon study teams, Objectively measured physical capability levels and mortality: systematic review and meta-analysis. BMJ.

[57] Harlow S.D., Gass M., Hall J.E., Lobo R., Maki P., Rebar R.W. (2012). Executive summary of the Stages of Reproductive Aging Workshop + 10: addressing the unfinished agenda of staging reproductive aging. Menopause.

[58] Bonaccio M., Di Castelnuovo A., Costanzo S., Ruggiero E., Esposito S., Cerletti C. (2025). Combination of a traditional Mediterranean diet with ultra-processed food consumption in relation to all-cause and cause-specific mortality: prospective findings from the Moli-sani Study. Clin. Nutr..

[59] Rothman K.J. (2008). BMI-related errors in the measurement of obesity. Int. J. Obes. (Lond)..

[60] Wells J.C., Fewtrell M.S. (2006). Measuring body composition. Arch. Dis. Child..

[61] Kuk J.L., Katzmarzyk P.T., Nichaman M.Z., Church T.S., Blair S.N., Ross R. (2006). Visceral fat is an independent predictor of all-cause mortality in men. Obesity (Silver Spring).

[62] Mozaffarian D. (2026). The 2025-2030 dietary guidelines for Americans. JAMA.

