# Supplementary material for: Mediterranean Diet Adherence and Ultraprocessed Food Consumption in Relation to Body Composition and Muscle Function Outcomes among Midlife and Older Women: NHANES 2007–2018
Source: Curr Dev Nutr. 2026 May 26;10(6):107726. doi: 10.1016/j.cdnut.2026.107726 (PMC13276543; doi:10.1016/j.cdnut.2026.107726)
Supplement: Multimedia component 1 [file mmc1.docx]

**Table S1. Food group definitions, variable derivation, and population-specific median cut-points used to construct the mMDS.**

| **Component** | **Variables** | **Source Files** | **Derivation** | **Unit** | **Median** | **Scoring** |
| --- | --- | --- | --- | --- | --- | --- |
| Fruits | DR1T_F_TOTAL, DR2T_F_TOTAL | FPED | Mean of two recalls | cup eq/day | 0.95 | ≥ median = 1 |
| Vegetables & potatoes | DR1T_V_TOTAL, DR2T_V_TOTAL | FPED | Mean of two recalls | cup eq/day | 1.61 | ≥ median = 1 |
| Whole grains | DR1T_G_WHOLE, DR2T_G_WHOLE | FPED | Mean of two recalls | oz eq/day | 0.71 | ≥ median = 1 |
| Legumes, nuts, seeds | DR1T_PF_LEGUMES, DR1T_PF_NUTSDS,  DR2T_PF_LEGUMES, DR2T_PF_NUTSDS, | FPED | Sum of legumes and nuts/seeds within each recall, then mean of two recalls | oz eq/day | 0.59 | ≥ median = 1 |
| Fish | DR1T_PF_SEAFD_LOW, DR1T_PF_SEAFD_HI, DR2T_PF_SEAFD_LOW, DR2T_PF_SEAFD_HI | FPED | Sum of seafood components within each recall, then mean of two recalls | oz eq/day | 0 | ≥ median = 1 |
| Meat | DR1T_PF_MEAT, DR1T_PF_CUREDMEAT, DR1T_PF_ORGAN, DR1T_PF_POULT,  DR2T_PF_MEAT, DR2T_PF_CUREDMEAT, DR2T_PF_ORGAN, DR2T_PF_POULT | FPED | Sum of meat components within each recall, then mean of two recalls | oz eq/day | 3.43 | < median = 1 |
| Dairy | DR1T_D_TOTAL, DR2T_D_TOTAL | FPED | Mean of two recalls | cup eq/day | 1.37 | < median = 1 |
| Fat ratio | DR1TMFAT, DR1TSFAT, DR2TMFAT, DR2TSFAT | FNDDS | Mean monounsaturated fat divided by mean saturated fat | ratio | 1.10 | ≥ median = 1 |

Food group intakes were calculated as the mean of two 24-hour dietary recalls. Median cut-points were calculated as survey-weighted medians within the analytic sample. Intakes were energy-adjusted to 2000 kcal/day using the density method, whereby each food group intake was divided by total energy intake (kcal/day) and multiplied by 2000 (i.e., [food group intake ÷ total energy intake] × 2000). The monounsaturated-to-saturated fat ratio was not energy-adjusted. mMDS = Mediterranean Diet Score; FPED = Food Patterns Equivalents Database; FNDDS = Food and Nutrient Database for Dietary Studies.

**Table S2. Derivation of UPF intake variables from NHANES dietary recall data based on NOVA classification.**

| **Variable** | **Description** |
| --- | --- |
| DR1T_Propgram_Novag4 | Proportion grams from UPF (Day 1) |
| DR2T_Propgram_Novag4 | Proportion grams from UPF (Day 2) |
| avg_G4_propgram | Derived mean of Day 1 & Day 2 |

UPF intake was defined as the proportion (0–1) of total grams per day derived from NOVA Group 4 foods, calculated as grams of ultraprocessed foods divided by total grams of all foods consumed per day (grams UPF / total grams). The final analytic exposure variable used in regression models was the mean proportion across two 24-hour dietary recalls (avg_G4_propgram). NOVA classifications were applied to NHANES food codes using an externally developed dataset based on previously published methods [24]. UPF = ultraprocessed food.
